# Sleeplessness—A Nervous Disease

**Published:** 1870-10

**Authors:** Ben. H. Pratt


### THE BISTOURY.

VOL. VI. ELMIRA, N. Y. OCT., 1870. No. 3

### Communications.

### SLEEPLESSNESS---A NERVOUS DISEASE.

BEN. H. PRATT, A. M.

The complaint of Sleeplessness, so frequently heard in our midst, is a disease—or rather, fast becoming such. Although not strictly set down in the department of Pathology at present, the day is not far distant when the Doctors will get it there, and all manner of specifics will be invented for its alleviation.

Prefatory to such an ultimatum therefore, I purpose a word or two with that great class of restless ones—the bed rollers—the mattress thumpers—the pillow butters—the sheet tanglers—the floor walkers—the legion of wide-awakes—and for whom we would prepare a common-sense anodyne in advance. The dose may not be agreeable to all—may be an impossible one for many—but will cure if no scruples are entertained about the dram, for there's not a grain of fatality about it. Nor is our soporific one of those terribly tangible occupyers of ounce vials, nor yet resides in peculiarly suggestive round boxes, nor is it done up in paper with much label of gilt; for, verily, these are the weapons of wise doctors—the shields of quackdom—the batons of charlatanism.

That Sleeplessness is the result of Nervousness is not sufficiently appreciated. Nervous ailings are too frequently ascribed to a deficiency of sleep; whereas the latter is the *effect* of the former, and not the *cause.* Hence, if undue nervousness can be avoided, refreshing, recuperating, healthful sleep must follow.

In these latter days, especially in populous communities, modes of living have become unnatural to a degree that utterly confounds the advocates of the old time *regime* and sets old fashioned folk to wondering what prevents the race from speedy extinction. So gradually do we glide from one absurdity to another—so imperceptibly abandon the ways our fathers trod and invent or adopt seemingly shorter and more agreeable paths to the goal of life—that before we become aware of our actual position, we find that life has become wholly bereft of the natural, and has assumed an aspect of artificiality almost too gross for realization. To be born and reared as were our ancestors, is a thing not to be thought of, except to ridicule. To be clad as they were, we deem preposterous beyond measure. To eat as they ate—what they ate—when they ate—these be vulgarities at which modern society elevates its nose, and recoils from with a highly genteel shudder. To dwell in the distressingly plain manner our much honored but cordially pitied progenitors dwelt, is so far beyond our conceptions as to provoke multiplied wonderment as to how they endured so much misery. It is truly affecting to ponder upon the life our dear old great-great-grandmother led, poor soul, who never was called for, poor thing, to attend the dear delightful opera, of a lovely moonlight eve, nor ever enjoyed the healthful, inspiriting, ecstatic delight of a run to Saratoga. Poor old G. G. grandma! alas! she's dead, say we, and never had a joy!

Now we wouldn't advise anybody to waste much sentiment, nor many tears over the aforesaid G. G. G.—besides, she's quite dead, and if her dear old soul is cognizant of your method of worrying out life, she pities you tenfold more than you ever can pity her. "Why?" We'll show you wherefore, anon.

LECTURE.—My dear Araminta and Augustus:—No, I beg pardon—it isn't quite fair to select such extremes of snobbery as representative men and women, even in this age of extremes. Let's modify it a trifle. My dear George and Eliza:—It was with sincere regret and unalloyed sorrow that I listened to the late recital of your maladies; uppermost among which, and most serious of all, is that of Sleeplessness. I assure you, my sympathy in your behalf is thoroughly awakened, and my anxiety in the matter is heightened, not so much because I cannot afford you relief—for that were easy were you entirely under my control—but because I doubt whether you sufficiently appreciate the moral strength and heroism necessary to adopt the treatment it is necessary to apply in your case. The difficulty of affording you a realizing sense of your situation is the greatest barrier to recovery I shall encounter in the path toward restoration. You are nervous—not naturally—not constitutionally—not chronically—but artificially, the result of certain modes of living, which, though not deemed injurious by your associates, nor open to censure from even the most prudishly inclined, are yet the prime cause of all the ills you suffer under. Fostered in the lap of luxury, you have known few wants that were not gratified—few wishes that were not realized—few enjoyments that were not supplied. Grown to maturer years, there have been few pleasures withheld from you, and though you have overstepped no bounds of propriety, you have done yourselves incalculable harm. Accustomed to realize all the legitimate pleasure to which society has afforded you access, and having become sated with what it ordinarily offers, you have essayed the realm of excitables. You do not so denominate them, but such they undeniably are. They lurk under pleasant exteriors and fascinating guises—come to you with the blandest, the most innocent faces, wearing no feature save of tranquility and repose. To rank them among the excitables seems to you strangely out of keeping, and here lies their danger. How frequently is nature violated in your every-day life? Your reason does not prompt you so well as the instinct of animals does them, otherwise you would rise betimes of a morning and avoid that fluttering your heart sets up when you rise late. This initiates the *nervousness* of the day. Then you eat, immediately, hurriedly, of a breakfast far too varied, far too rich, far too stimulating, which adds materially to the shock your nerves have already experienced. You then plunge into your business, of whatever kind it may be, pell mell, heedless of a twinge or two of bowels or head. Your mid-day meal is, if possible, a greater outrage upon common sense and a bitterer enemy of your digestion than the former one, and a brisk walk thereafter does not mend matters a bit. Before nature can come to your relief you try your best to thwart her by forcing down a supper, which, even if light, you suffer from and try to forget amid the excitement of your club, or perhaps by a violent political discussion, or solving a knotty business problem, my dear George; or by a tirade against servants and the miseries of housewifery, my dear Eliza; or perchance you accept the kindly hospitality of a neighbor or friend and steal some hours from the province of sleep, retiring late, tired, nervous to a degree that if it does not banish sleep, robs it of its normal re-creating power, and leaves you worse prepared for another day than you were for the last. A repetition of the above from day to day, with occasional extras of sleep-robbing, nerve-shattering excitements, brings about a final condition of body and mind, under which it is simply preposterous to expect that sleep, the sleep of nature, shall woo you and invigorate as well. Mother nature is a just and strict disciplinarian, and shows her children no partiality. The naughty ones must suffer for their disobedience in every particular. The little world you live in may not, does not call you a bad lot, and Mrs. Grundy may even point you out as a model pair; but your little world and the Grundy family too, are incompetent judges of your cases, and err reprehensibly themselves. And now, since you have consulted me in this matter, forgive me if I offer you a prescription; and this, not after the manner of Doctors, curiously disguised in horridly mutilated latin, and diversified with blind hieroglyphics which would puzzle old Æsculapius himself, were he to resurrect his much abused self; but in as fair an idiom and as square anglo-saxon as my profound appreciation of common sense will allow. Take of Honesty—the best your market affords—all you can gather; add thereto a Conservative Diet gleaned from the cuisine of a century back—Prof. Blot to the contrary notwithstanding; with the above mingle as generous a quantity of Early Retiring and Rising-with-the-Sun as your occupation will allow; season the above plentifully with Innocent Amusement and Recreation, carefully selected, and mix the whole around your hearthstone in the presence of your family and a very few chosen associates. Continue the dose until you observe an alterative effect, after which, I think, you will conclude that you can't keep house without hourly indulgence in this gratuitous compound.

To this I wish to append some explanatory remarks, in order that the best effects may arise from the adoption and use of my formula. Of the first ingredient, Honesty, be cautious where you procure it. There is much of the spurious article in the market. Don't trust the opinions of friends even—few are good judges of the commodity. Test it in the crucible of conscience. The policy of honesty will not answer—it's too thin—it won't wash—go for the genuine. Humble pie will be found an excellent diet, especially during the transition state from your present regimen to that I propose. You will find the Early Rising ingredient probably as disagreeable as any, but if it be gulped at one draught, and not sipped, the effect will be palatable rather than otherwise. Among the amusements I would specially commend is the very novel and interesting game called Domestic Felicity, and is adapted to any number of players. Very few are acquainted with this admirable pastime, so you may possibly get no instructions from your neighbors. You can readily master it, however, after a few trials, and you will find it one of the most admirable methods of becoming intimately acquainted with your wife or husband and the family generally, a result which is seldom attained now-a-days. This acquaintance it is well to keep up assiduously, for much good may result from it some day. These trifling suggestions, added to many innovations which your own ingenuity will readily suggest, are, I think, ample for the present. For your better satisfaction, and to promote confidence in my recipe, I will merely state, that "in no case where it has been fairly tested and used according to the directions, has it ever failed to produce the desired effect and bring about a perfect cure." Under such circumstances I am safe in warranting my remedy. I may add that "it is a safe, certain and speedy cure." "It's effects are magical"—"an unfailing remedy"—"no form of nervous disease (the great first cause of sleeplessness,) fails to yield to its wonderful power"—"a few days affords the most astonishing relief"—"it contains no mercury"—"it has the unqualified approval of the best physicians"—"it is *not* sold by all dealers in drugs and medicines"—&c., &c., &c.

But, my dear George and Eliza, I know you so very intimately—am so well acquainted with your little foibles and failings—I am almost certain that you will feel ever and ever so grateful to, and will spend a volume of thanks and a great wealth of gratitude upon me for what you will term my kind services in your behalf; but you won't take my advice nor test my prescription. I *know* you will not; and I know too, *why* you will not. You'll sit down—you two—and wonder first, what Mrs. Grundy will say—then what Aunt and Uncle will think—then what your neighbors will do—then, how you can make the sacrifice yourselves—then weigh the considerations together, pro and con—and the upshot of the matter will be, you won't attempt the cure. You'd rather go on and on—twisting, turning, rolling in your uneasy beds—thinking against your desire to think—provoked beyond measure because, forsooth, you can't command your limbs to rest, nor your body to repose, nor your eyelids to sleep. Wretched indeed are ye, but the present is scarcely a tithe of what's to come. Aged before your time—fretful and peevish by reason of premature age—you'll hobble and limp adown the latter pathways of life, a burden to yourselves and a care to those who, but for your earlier mistakes, might be your companions instead of your nurses. Boy fashion, the plum of to-day is preferable to the plum-pudding of to-morrow, all the world over, but when that morrow comes, with it will come the useless wish that you had denied yourselves a little yesterday. Sleepless nights have done much toward peopling our asylums—much toward lengthening our mortuary lists—much toward lining the pockets of Doctors; and is making daily victims of far too many of us—victims of this, that and the other ills with which the world abounds.

The *disease* of sleeplessness—for such it has assuredly become—is one of comparatively late date, and the prevalence of it is alarming to those who have investigated into the number of those subject to it. In the modern apportioning of duties, sleep is almost entirely left out of the reckoning, whereas the majority of human kind actually require eight hours for undergoing that recuperation which sleep alone can effect. And not only this, but Nature has so ordained that she will not accept the spurious coin of morning naps for the pure jewel of sound sleep, which only comes between dark and daylight. Nor will she be long tampered with. The world will soon learn to its sorrow that it must either return to first principles, or reap the consequences with which Nature surely visits the violaters of her perfect laws.

---

WHITE WASH THAT WILL NOT RUB OFF.—Mix up half a pailful of lime and water; take half a pint of flour and make a starch of it, and pour it into the white-wash while hot. Stir it well, and make it ready for use.

